# Ensemble localized patch residual convolution neural networks for pneumonia detection using chest X-ray

**DOI:** 10.1186/s12911-026-03497-y

**Published:** 2026-04-27

**Authors:** Jyotidip Barman, Anil K. Giri

**Affiliations:** 1 Institute for Molecular Medicine Finland (FIMM), Helsinki Institute of Life Science, University of Helsinki, Helsinki, Finland; 2 Molecular and Cellular Systems of Life Doctoral Programme, Faculty of Medicine, University of Helsinki, Hartmaaninkatu 2, Helsinki, Finland; 3 Foundation for The Finnish Cancer Institute (FCI), Tukholmankatu 8, Helsinki, 00290 Finland; 4 iCAN Digital Precision Cancer Medicine Flagship, University of Helsinki, Helsinki, Finland; 5 HiLIFE-Institue for Molecular Medicine Finland, Biomedicum 2U, Tukholmankatu 8, Helsinki, 00290 Finland

**Keywords:** Pneumonia, Chest X-ray, Deep learning, Residual network, CNN

## Abstract

**Background:**

Pneumonia is a life-threatening disease, especially in infants and older individuals. Chest X-ray is the most common method for the detection of lung inflammation especially when the causes of pneumonia are unknown. However, the evaluation of X-ray images requires special human expertise hence, the detection process can be error-prone and is also time- and resource-consuming. Automating the pneumonia-detection process from chest X-ray using a computer algorithm will improve precision, save time and resources.

**Methods:**

In this manuscript, a deep learning strategy called an ensemble of residual neural networks (eRes-NET), is presented for efficient pneumonia detection using X-ray images of the chest. The eRes-NET learns high-level features from multiple CNN models trained on smaller patches (25 × 25 pixels) of an image to identify distinguishing features of infection. In addition, to tackle the inadequate contrast of chest X-ray images leading to ambiguous diagnosis, the contrast-limited adaptive histogram equalization (CLAHE-DWT) technique is used to pre-process the images before pneumonia prediction.

**Results:**

Across a cohort of 8,266 chest X-ray images, eRes-NET achieved 93% accuracy and 94% AUROC on an independent posteroanterior test dataset. The approach performed favorably against 13 published methods (exceeding 8 under their reported protocols), noting differences in datasets and evaluation procedures.

**Conclusions:**

The proposed eRes-NET approach showed strong performance for pneumonia detection using chest X-ray images and may support efficient automated screening.

**Supplementary Information:**

The online version contains supplementary material available at 10.1186/s12911-026-03497-y.

## Background

Pneumonia is inflammation of the lung mostly instigated by bacteria, viruses, or fungi infection [1]. It can be life-threatening, especially in infants, old-age adults (> 65 years), and individuals suffering from chronic diseases like asthma or diabetes [2]. It kills nearly 0.8-2 million children under the age of 5 annually [3]. Chest radiography is one of the most routine and established forms of radiological investigations to diagnose the level of inflammation in the lung (called infiltrates). However, the diagnosis of pneumonia by chest X-rays needs expert radiotherapists for evaluation [4]. However, the assessment varies from expert to expert and is time and resource consuming. Further, it is error-prone due to the often-subtle presentation of pneumonia as the appearance of pneumonia in an X-ray can be hazy and misapprehended with other diagnoses. Thus, an automatic method for pneumonia detection can be advantageous for treating the infection without any delay, predominantly in resource-limited settings in low and middle-income countries.

Recently, there has been increasing interest in the application of “Deep Learning” strategies for the identification of pneumonia using Chest-Xray data. Different variants of convolutional neural networks (CNNs) for pneumonia detection in chest X-rays have been proposed using multiple datasets. For example, Rajpurkar et al. (2017) [5] developed a DenseNet-121 layer-based deep-learning architecture named ChexNet to detect 14 diseases from a lot of 112,200 chest X-ray images from the OpenI dataset. Although many of the models have been highly accurate in predicting pneumonia infection in public datasets [6]. However, they still have limitations in generability and sensitivity and are not advanced enough to replace physicians in medical diagnosis hence, they need improvement as it can assist in finding signs of pneumonia from the chest X-rays. Vikash Chouhan et al. [7] showed one of the most impressive results with an ensemble of pre-trained models (e.g. Inception V3 model, DenseNet) while Zhang D et al. [8] showed that pre-processing and image enhancement techniques, such as dynamic histogram equalization improve results.

In this context, an ensemble of multiple convolutional network models has emerged as a promising strategy to enhance the performance and robustness of CXR interpretation systems [9–11]. However, in most cases, the entirety of the scan is processed by the models to classify the anomaly in the scan. However, extraneous sections of the image can act as blockers or noise, hindering the model’s predictive capabilities [12]. By selectively focusing on relevant patches, the model can mitigate the influence of such distractions and improve the accuracy of its predictions [13]. Having these localized patch networks allows an accurate prediction and visualization of the pneumonia-related anomalies in the scans. Ensembling multiple such patch-level models (each specialized in detecting pneumonia in its local region) further boosts overall performance while reducing the impact of any single patch’s misprediction [14].

This study proposes a novel ensemble of residual-deep learning network models for pneumonia detection which combines the predictions made by multiple convolution networks trained on pneumonia-relevant patches in the images. Additionally, since the miniature patch networks are only working towards finding anomalies in the relevant sections of the X-ray, it reduces the number of training parameters, and time to build complex convolution networks required for deep feature extraction. To counter-balance the issue of vanishing gradient problems commonly seen during model training, we have also implemented residual learning in the model. The model has been trained on the Guangzhou Women and Children’s Medical Center chest X-ray dataset [15], and validated on the pneumonia and normal Chest X-ray PA dataset [16]. The proposed model attained a 93% accuracy on pneumonia detection in the Chest X-ray PA dataset.

## Methods

### Datasets

In this study, we utilize a dataset comprising 8266 images consisting of 5400 pneumonia and 2866 normal (non-pneumonia) images in total from 2 datasets (Table 1).

**Table 1:** Distribution of the total data considered in our study

Dataset	Total Sample Count	Pneumonia Samples	Normal	Ref
Guangzhou Women and Children’s Medical Center chest X-ray dataset	5216	3875	1341	[15]
Pneumonia and Normal Chest X-ray PA Dataset	3050	1525	1525	[16]

The Guangzhou dataset count used here (5,216 images) reflects only the training portion of the public dataset, excluding the original test subset (624 images) provided by Kermany et al. [15]. The PA Chest X-ray dataset (3,050 images) was used exclusively for independent evaluation, ensuring that our final test set was entirely separate from the training data

**Guangzhou Women and Children’s Medical Center chest X-ray dataset** We trained our model on 5216 chest X-ray images (anterior posterior) from a pediatric patient’s cohort (1–5 years old) from Guangzhou Women and Children’s Medical Center, Guangzhou [15]. The data is publicly available and was downloaded from the Kaggle website (https://www.kaggle.com/datasets/paultimothymooney/chest-xray-pneumonia). The Guangzhou dataset consists of 3875 pneumonia images and 1341 normal images and represents only the training portion of that publicly available dataset. We excluded the original test subset, as our study evaluates on an external test set (Table 1).

**Pneumonia and Normal Chest X-ray posteroanterior dataset** Additionally, we analyzed 3052 posteroanterior (PA) chest X-ray images of 1525 pneumonia cases, and 1525 normal individuals to test the prediction ability of our model. The images have been collected by Amanulla et al. [16] from different sources (e.g. NIH) and have been deposited at the Mendeley data repository (https://data.mendeley.com/datasets/jctsfj2sfn/1). The data is widely used for the development of image-based prediction models.

### Workflow of the present model

**Fig. 1:**
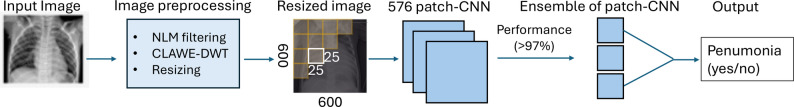
Schematic diagram for the prediction model. A flow diagram of the proposed methodology. The input chest X-ray (250 × 250) undergoes preprocessing, including denoising (Non-Local Means filtering) and contrast enhancement (CLAHE-DWT). The image is then resized to 600 × 600 pixels, divided into 25 × 25-pixel patches, and classified using an ensemble of CNN-based patch classifiers. The highest-performing patch models (> 97% accuracy) contribute to the final pneumonia vs. normal classification. Each preprocessed CXR was resized to 600 × 600 and partitioned into a fixed 24 × 24 grid (576 patches; 25 × 25 px). Patches inherited the parent image label; no manual patch mining was applied. All splits were performed image-wise so that patches from the same image never appeared across train/validation/test (to prevent leakage). At inference, we averaged probabilities across all 576 patches to obtain a single image-level score and thresholded it for the final label. Among the tested configurations, 25 × 25 patches and 600 × 600 inputs provided the best validation accuracy and AUROC trade-off (see Supplementary Tables 1–2) and were adopted as default

All images were included through each preprocessing step — no images were removed or filtered out manually. A flow diagram of the proposed methodology is shown in Fig. 1. The model prepossesses the input image (250 × 250) data by denoising it is using the Nonlocal means (NLM) filtering algorithm [17], enhancing the contrast by contrast limited adaptive histogram equalization discrete wavelet transform (CLAHE-DWT) approach [18] and resizing the image to 600 × 600 pixels. Next, it extracts patches of 25 × 25 pixels size from the resized images (a total of 576 patches per image).

All patches inherit the same label as their source image – patches from a pneumonia-positive X-ray are labeled ‘pneumonia,’ and patches from a normal X-ray are labeled ‘normal. We selected a 600 × 600 pixels image size as input and 25 × 25 pixel patch sizes for training the model as they provided maximum accuracy for the model in comparison to other pixel sizes (Supplementary Tables 1 and 2).

After that, it trains a CNN over the image patch, and the patch CNN ultimately votes on the image’s label. In other words, the model trains on image patches but still aims to classify the entire image as pneumonia-positive or normal. All splits are image-wise (no patches from the same image appear in both train and validation/test). At inference, we average the 576 patch probabilities to obtain a single image-level score and threshold it for the final label. Briefly, the CNN model first augments the image data using the Keras’ Image Data Generator. The Keras Image Data Generator performs random rotations, shifts, and flips so that at each epoch the model effectively sees new variations of the images. Then, the model trains on these augmented patch images and classifies the image as a whole into benign or pneumonia positive. These patch CNN models with performance accuracy > 97% are selected and used to develop ensemble models (Supplementary Table 4). The ensemble model is retrained and fine-tuned, and the output of the ensemble model is the final prediction for pneumonia or normal. Both the patch CNN and ensemble model have been trained for 50 epochs.

### Residual-network-based architecture

**Fig. 2:**
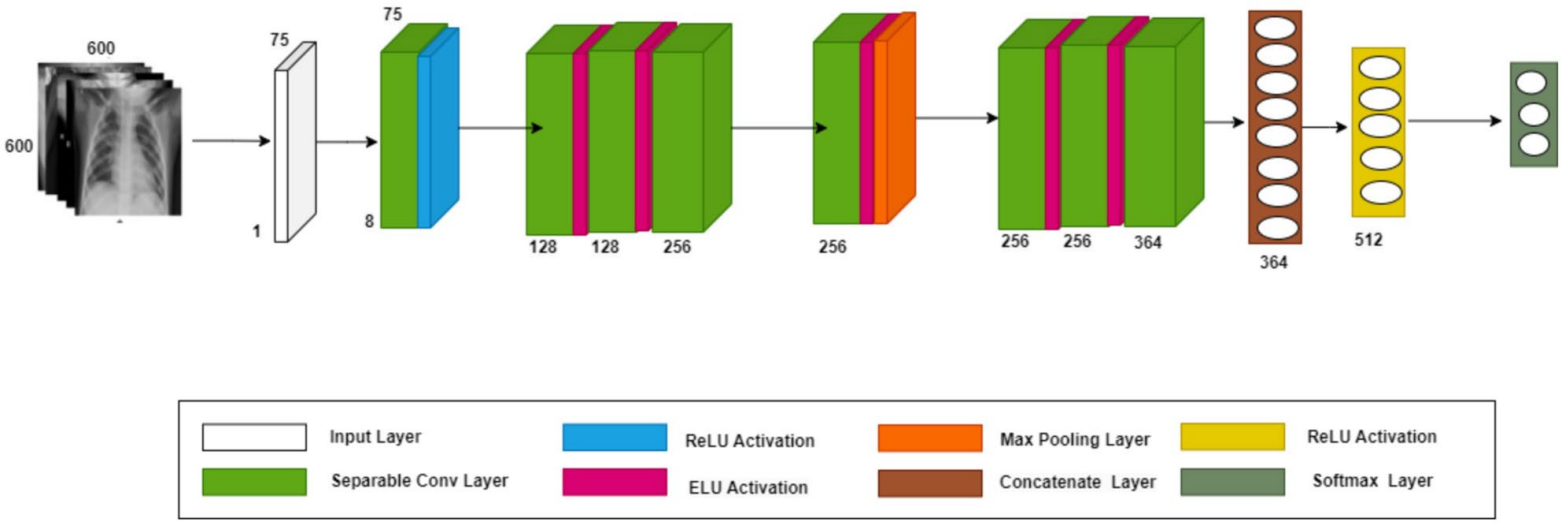
Detail diagram for the prediction model. The image showcases a single convolutional neural network (CNN) architecture branch for simplicity

The image (Fig. 2) showcases a single convolutional neural network (CNN) architecture branch for simplicity, even though we have implemented two branches with the same components. In the model, the initial processing begins with a convolutional layer named “initial layer consists of 1 SeparableConv2D layer, 1 BatchNormalization layer, and 1 RELU activation layer. It then passes through five residual blocks, each incorporating SeparableConv2D layers, batch normalization, and skip connections. The final classification layer outputs a probability score for pneumonia vs. normal. The 2 branches together consist of 120 layers. The outputs from the two branches are concatenated using a concatenated layer. Next, a ReLU activation of the concatenated output followed by a softmax will provide the predicted output.

### Hyperparameter tuning

We used a manual grid search [19] algorithm to optimize the hyperparameter of the models in this work as done before in our manuscript [20]. We chose this method because it helps assess how the model performs unseen data from different image regions. We adjusted the learning rate using the ReduceLROnPlateau callback function [21] in the Keras package based on prediction accuracy during training. We used ReduceLROnPlateau because it is a state-of-the art optimization technique that decreases the learning rate when the specified performance metric stops hence allowing the model to escape local minima and improve further. Thus, we kept the learning rate the same as long as it improved the metric quantity, but the learning rates were reduced when the results stagnated.

In our implementation, we additionally combine ReduceLROnPlateau with a Root Mean Square Propagation-based optimization algorithm. Dropout Regularization with a dropout rate of 0.5, an initial learning rate of 0.001, and a batch size of 32 to prevent overfitting randomly dropping neurons during training, hence preventing complex co-adaptations with one another. Further, we optimized the hyperparameters for model architecture defined as the number and types of layers (e.g. Conv2D, Dense, etc.) using their configurations.

The model comprises two identical branches, each built from a SeparableConv2D–BatchNorm–ReLU stem followed by 5 residual blocks (3 × 3 kernels; feature maps 32→64→128), then global average pooling. The branch outputs are concatenated and passed through a 128-unit dense layer (ReLU) and a 2-way softmax. Full layer specifications (type, filters, kernel/stride, activation, parameters) are provided in Supplementary Table 5.

For hyperparameters optimization, initially, we used a simple train-test split where we allocated 75% of the original data for training and 25% for testing. In this approach, we initially trained the model with random hyperparameters on the training split and evaluated the resulting model on the test split. Based on the evaluation, the algorithm selects new hyperparameters. The process is repeated till the minimum loss is achieved. The best hyperparameters (4,967 trainable out of 59,550,177) were chosen based on the evaluation metrics from the test set. We used cross-entropy loss to measure the magnitude of error between predicted and actual diagnosis.

As cross-entropy loss can underperform other loss functions, especially with imbalanced class data as it can get biased toward the majority class, we randomly undersampled pneumonia cases so that each mini-batch contained a 1:1 ratio of pneumonia to normal patches in each epoch [22]. Further, we augmented both classes to mitigate any loss of information from under-sampling. We also augmented the image data using Keras’ ImageDataGenerator to further enhance class balance and prevent overfitting. These included random rotations (± 15 degrees), horizontal flips, random zooms (up to 10%), and random shifts (up to 10%). This balanced mini-batch strategy forces the model to learn from both classes equally.

Further to ensure proper training of the model, we performed a 5-fold cross-validation of the model in the Guangzhou Women and Children’s Medical Center dataset to examine the robustness in the performance of the model. We used the sklearn.model_selection module in Scikit-Learn library to ensure the balanced class distribution (i.e., pneumonia vs. normal cases) across each fold. Briefly, the dataset was divided into 5 equally sized subsets (folds), each preserving the original label proportions. In each iteration, 4 folds were used for training the model and 1-fold was held out for validation. This process was repeated five times so that each fold served as a validation set once. For each fold, the model was initialized from scratch and trained for up to 50 epochs, with early stopping and learning rate scheduling applied to avoid overfitting.

All experiments were conducted on an NVIDIA A100 GPU. The full 5-fold cross-validation procedure required approximately 14 h in total (mean ± SD: 2.8 ± 0.4 h per fold). For inference, the model processed a batch of 576 image patches in less than one second on the GPU. The final trained model has a compact memory footprint of approximately 9 MB. To ensure reproducibility, all timing measurements were performed using scripts provided in the publicly available github repository.

**Performance matrix**:We used binary cross-entropy loss function to train the models because our data contained normal and pneumonia classes. Further, to optimize the parameters we used the Adam optimizer approach. We evaluate our models with six metrics commonly used in binary class prediction problems: accuracy, precision, recall, area under the curve (AUC), and F1 score. We considered all these parameters together to assess the performance of the model, instead of focusing on only one stand-alone metric. When the model classifies an image, it could be true positive (TP), true negative (TN), false positive (FP), and false negative (FN). TP shows accurately predicted positive instances, TNs are correctly classified as negative instances.

Accuracy is estimated as the number of correctly classified predictions out of a total number of predictions and is defined as

$$\:\mathrm{A}\mathrm{c}\mathrm{c}\mathrm{u}\mathrm{r}\mathrm{a}\mathrm{c}\mathrm{y}=\mathrm{T}\mathrm{P}+\mathrm{T}\mathrm{N}/(\mathrm{T}\mathrm{P}+\mathrm{F}\mathrm{P}+\mathrm{T}\mathrm{N}+\mathrm{F}\mathrm{N})$$

Precision is the number of correctly classified positive predictions from the total predicted both true and false positives and can be statistically defined as

$$\:\mathrm{P}\mathrm{r}\mathrm{e}\mathrm{c}\mathrm{i}\mathrm{s}\mathrm{i}\mathrm{o}\mathrm{n}=\mathrm{T}\mathrm{P}/(\mathrm{T}\mathrm{P}+\mathrm{F}\mathrm{P})$$

The recall is the score of true positive predictions to the instances that belong to the positive class.

$$\:\mathrm{R}\mathrm{e}\mathrm{c}\mathrm{a}\mathrm{l}\mathrm{l}=\mathrm{T}\mathrm{P}/(\mathrm{T}\mathrm{P}+\mathrm{F}\mathrm{N})$$

F1 score is a performance metric averaging both precision and recall of a model and is estimated as:

$$\begin{aligned}\:\mathrm{F}1-\mathrm{s}\mathrm{c}\mathrm{o}\mathrm{r}\mathrm{e}=&2\times\:\mathrm{p}\mathrm{r}\mathrm{e}\mathrm{c}\mathrm{i}\mathrm{s}\mathrm{i}\mathrm{o}\mathrm{n}\times\:\mathrm{r}\mathrm{e}\mathrm{c}\mathrm{a}\mathrm{l}\mathrm{l}\mathrm{p}\mathrm{r}\mathrm{e}\mathrm{c}\mathrm{i}\mathrm{s}\mathrm{i}\mathrm{o}\mathrm{n}\cr&+\mathrm{r}\mathrm{e}\mathrm{c}\mathrm{a}\mathrm{l}\mathrm{l}\end{aligned}$$

We also used the area under the receiver operating characteristic (AUROC) curve to evaluate the performance of our pneumonia classification model. The ROC curve plots the true positive rate and the false positive rate to show the trade-off between the two axes at various thresholds.

**Comparison of the model with other published models** Next, we also compared the performance of our model with the other published deep learning models for pneumonia detection using the chest X-ray data (23–34) for their performance using the area under the receiver operating characteristic curve.

## Results

### Model training and testing

We first developed the model on the Guangzhou Women and Children’s Medical Center chest X-ray dataset from the Kaggle repository. We randomly sampled 75% of the full data for training purposes and used the remaining 25% for testing and hyperparameter tuning.

We first used a single hold-out split of the Guangzhou Women and Children’s Medical Center Chest X-ray dataset for quick hyperparameter prototyping. We then performed 5-fold cross-validation on the whole training dataset to assess robustness and finally reported generalization on a fully independent posteroanterior (PA) dataset.

Across 5 folds (Guangzhou), performance was mean ± SD: Accuracy 99.97% ± 0.0447%, AUROC 0.99978 ± 0.000179, Precision 99.97% ± 0.0358%, Recall 99.97% ± 0.0358% as shown in Table 3.

During training, we monitored performance on a test split of the Guangzhou dataset, which was only used for hyperparameter tuning. However, the final performance was strictly evaluated on a completely independent test dataset (Pneumonia and Normal Chest X-ray PA dataset), ensuring no overlap with the training or validation phases.

**Fig. 3:**
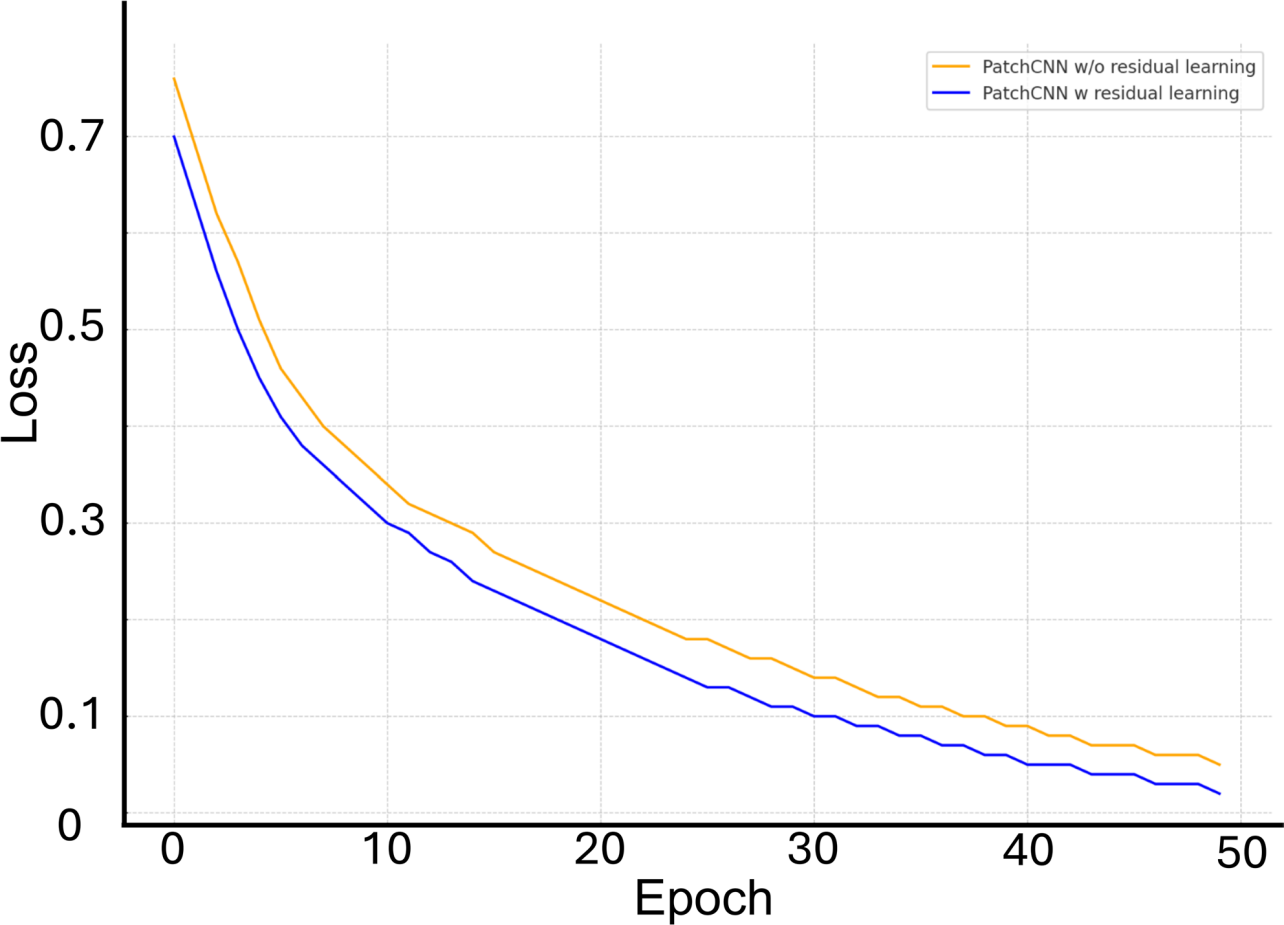
Training convergence with and without Residual Learning: A line plot shows the proposed methodology’s binary cross-entropy loss with and without residual learning

**Fig. 4:**
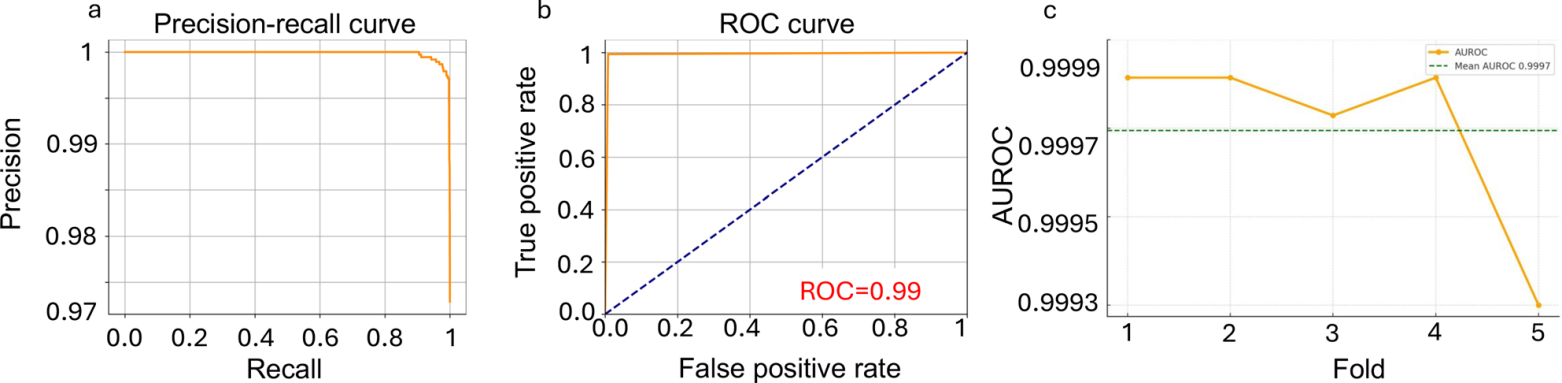
Performance evaluation of eRes-NET. (**a**) Precision-Recall curve, showing a high precision Precision–Recall curve and (**b**) ROC curve for eRes-NET on the Guangzhou validation set (**c**) Line-plot showing AUROC for the 5-fold cross-validation on the Guangzhou validation set

We observed a lower loss level with a residual learning framework as compared to without it while training (Fig. 3). Next, we predicted the accuracy of the trained model in predicting pneumonia in 25% of the pneumonia Guangzhou Women and Children’s Medical Center chest X-ray dataset. The results are based on the average of predictions from an ensemble of models with performance higher than 97% accuracy for 50 epochs during a training run. An ensemble approach enhances performance without increasing training overhead and offers better generalization for model predictions. The precision-recall curve and receiver operating curve for the model during predicting pneumonia in the test set have been shown in Fig. 4. In the test set from the Guangzhou Women and Children’s Medical Center chest X-ray dataset, the model showed an accuracy and precision of 99% in detecting pneumonia (Table 2).

**Table 2:** Performance of the model in the test set from the Guangzhou Women and Children’s Medical Center chest X-ray dataset

Performance metrics	Grid search	5-fold cross validation
Accuracy(ACC)	0.99	0.99
Precision(P)	0.99	0.99
Recall(SE)	0.99	0.99
Specificity(SP)	0.99	0.99
F1 score	0.99	0.99
AUROC score	0.99	0.99

The average of the scores after 5 fold cross validation has been shown

To check the stable and robust performance of the model, we performed 5-fold cross-validation of the model using different random seeds and splits from the Guangzhou dataset (Table 2, Supplementary Table 3). The model achieved a mean accuracy of 99.7% (± 0.6) across multiple runs, which is in line with the reported performance of the model. The model’s precision (positive predictive value) at high recall levels is near 1.0, indicating very few false positives when nearly all true pneumonia cases are detected. In this test set, the model showed 99% accuracy and 99% positive predictive value (precision) in detecting pneumonia (as summarized in Table 2).

### Model validation on independent test set

Although the prediction results in the test-split sets reveals a model’s general behavior and learning capacity, they alone are insufficient for a comprehensive evaluation of model performance because the results can be too optimistic. This is partly because the test set has a similar origin, structure, and bias as the training data. Therefore, to further test the robustness and usefulness of our model to predict pneumonia, we tested its performance in a new, independent validation dataset called the Pneumonia and Normal Chest X-ray PA Dataset [16]. The validation set does not include any images reported in the train-test split sets of the Guangzhou Women and Children’s Medical Center chest X-ray dataset. Our model predicted an accuracy score of 93% and an AUROC score of 94%.

Next, we also compared the performance of our model with the other published deep-learning-based models for pneumonia prediction [6]. Our model outperformed 8 out of 13 tested models and provided a 93% accuracy for pneumonia detection in totally independent validation datasets indicating a practical utility of our model for pneumonia detection (Table 3). We have compared our model against published model using the reported performance statistics instead of re-implementations because differences in dataset composition, splits, and training protocols limit strict head-to-head conclusions. We therefore report these numbers as contextual rather than definitive rankings.

**Table 3:** Performance comparison of eRes-NET against other published pneumonia detection models, evaluated exclusively on an independent test dataset (Pneumonia and Normal Chest X-ray PA dataset)

Authors	Methodology	Performance
Chouhan V et al. 2020 [7]	AlexNet, InceptionV3, ResNet18, DenseNet121 and GoogLeNet	AUC = 0.99
Irfan A et al. 2020 [23]	ResNet-50, Inception V3, DenseNet121	AUC = 0.71
Jain R et al. 2020 [24]	VGG	AUC = 0.99
Togaçar M et al. 2020 [25]	Deep feature CNN	AUC = 0.99
César Ortiz-Toro et al. 2022 [26]	KNN based models	AUC = 0.83
Wang C et al. 2023 [27]	Graph-based CNN	AUC = 0.89
Motamed S et al. 2021 [28]	GAN	AUC = 0.89
Elshennawy NM et al. 2020 [29]	K-means, logistic regression	AUC = 0.60
Varshni D et al. 2019 [30]	DenseNet169 and SVM	AUC = 0.80
Malygina T et al. 2019 [31]	DenseNet-121with CycleGAN	AUC = 0.98
Acharya AK et al. 2020 [32]	Deep Siamese Network	AUC = 0.95
Zhou B et al. 2018 [33]	ResNet and CNN	AUC = 0.67
Ayan E et al. 2019 [34]	Xception, VGG16	AUC = 0.87
Our model	CNN	AUC = 0.94

AUC: Area under the curve; GAN: Genrative Adversial Network

## Discussion

By denoising the image, enhancing the contrast, resizing the image, and using an ensemble of multiple residual network-based CNN models trained on numerous smaller patches of X-ray data, we successfully detected pneumonia from the X-ray image with 93% accuracy and area under the receiver operating curve score of 94% in unknown Pneumonia and Normal Chest X-ray PA data. This success can be attributed primarily to the advanced features provided by (i) ResNets, which represent a significant breakthrough in deep neural networks where skip connections are deployed to mitigate the vanishing gradient problem, a common issue encountered in deep learning, (ii) denoising images, (iii) image enhancement technique to enhance image contrast without washing out appearance or causing problems like checkerboard affects the image, and (iv) training the model on smaller patches of the image which provides a nice regularization by estimating a smaller number of good parameters across many regions of individual image, and multiple regions of all other training images.

To illustrate the performance of our proposed model, we compared it against 13 published deep-learning and graph neural networks based on the latest methods reported in the literature for pneumonia prediction in literature, showing competitive performance exceeding the AUC of 8 out of 13 published models (Table 3). The source codes, along with the data and user descriptions are available on GitHub (https://github.com/jyotidiplearning99/CXR_Ensemble_Model ) for reuse. Furthermore, such an open-access algorithm can further help improve the accuracy of radiology-based disease prediction algorithms. However, we note that these comparisons are context-dependent – differences in training data and evaluation protocols across studies can influence reported performance. While our model achieved about 93% accuracy on the independent test set, suggesting strong potential, we caution that this result is within a controlled experimental setting. The model’s real-world utility would need to be confirmed on truly novel data from other hospitals or patient groups.

Despite the high performance observed, our study has certain limitations. First, the model was trained primarily on a pediatric dataset and tested on another curated dataset; its performance on other populations (e.g., adult patients or images from different hospitals) remains unvalidated. Second, our patch-based approach inherits image-level labels for all patches – this can introduce label noise, as not every patch from a pneumonia case is pathological. Although our ensemble strategy mitigates this by focusing on high-accuracy patch models, some reliance on global image context might occur. Third, both the training and test sets are from research repositories, which may not capture the full variability of clinical data (e.g. different X-ray machine settings, or interference from other comorbidities). This could limit direct clinical translatability. Further, in our approach some normal patches in positive CXRs are mislabeled because patches inherit image-level labels. Multiple-instance learning with attention pooling and class activation maps (CAMs)-guided soft labels can be integrated to mitigate patch-level noise and improve interpretability.

Finally, our method’s complexity (e.g. training multiple models and ensembling them) means increased computational overhead for real-time use. Hence, future prospective studies in clinical settings would be valuable to establish the model’s generalizability and practical utility. In clinic, false negatives cases (pneumonia infections identified as normal) risk delayed treatment, escalation of infection, whereas false positives cases (normal cases identified as pneumonia infection) may prompt unnecessary use of antibiotics or imaging. Thresholds can be tuned to favor high sensitivity cases under the guidance of radiological experts. We provide an image-level confusion matrix for the PA test set (Supplementary Table 5) and recommend task-specific threshold selection.

Further, in our study training of the model on a pediatric-dominant cohort (Guangzhou Women and Children’s Medical Center Chest X-ray dataset) and testing on a mixed-age PA dataset may introduce susceptibility towards age and demography-related factors (e.g. Geographic location), which can affect performance of the model in a newer cohort. Hence, they may limit the generalizability of the findings. Future work will include adult-only external validation and domain-adaptation analyses. Several recent architectures also report strong CXR performance (34–42); our cross-paper comparisons are therefore contextual rather than definitive, and head-to-head evaluations on shared protocols are warranted.

In the future, we will continue the develop more accurate classification architectures to diagnose different types of pneumonia by characterizing finer details of lung infection using other image types (e.g. CT-scan) which are otherwise difficult to diagnose using plain X-ray images. The demonstrated robust performance of our model across the different datasets suggests that our residual-network-based approach can be modified and applied to other datasets (e.g. CT-scan) from clinics to diagnose pneumonia and other diseases.

## Conclusion

In conclusion, this study demonstrates that a carefully designed ensemble of residual network-based CNN models, combined with targeted preprocessing techniques such as denoising, contrast enhancement, and patch-based training, can achieve strong performance in pneumonia detection from chest X-ray images. With an accuracy of 93% and an AUC of 94% on an independent test set, our approach highlights the effectiveness of leveraging ResNet architectures and localized feature learning to improve diagnostic prediction.

## Supplementary Information

Below is the link to the electronic supplementary material.

Supplementary Material 1

## Data Availability

The datasets analyzed during the current study are publicly available in the Kaggle repository, Chest X-Ray Images (Pneumonia), at[https://www.kaggle.com/datasets/paultimothymooney/chest-xray-pneumonia] and in the Mendeley Data repository, COVID19, Pneumonia and Normal Chest X-ray PA Dataset, at [https://data.mendeley.com/datasets/jctsfj2sfn/1]. The source code used in this study is publicly available in the GitHub repository, CXR_Ensemble_Model, at [https://github.com/jyotidiplearning99/CXR_Ensemble_Model].

## Abbreviations

AUROC: Area under the receiver operating characteristic curve
CAM: Class activation map
CLAHE-DWT: Contrast-limited adaptive histogram equalization–discrete wavelet transform
CNN: Convolutional neural network
CXR: Chest X-ray
FN: False negative
FP: False positive
NLM: Non-local means
PA: Posteroanterior
TN: True negative
TP: True positive
