# Supplementary material for: Ensemble localized patch residual convolution neural networks for pneumonia detection using chest X-ray
Source: BMC Med Inform Decis Mak. 2026 Apr 27;26:213. doi: 10.1186/s12911-026-03497-y (PMC13267658; doi:10.1186/s12911-026-03497-y)
Supplement: Supplementary file 1 — Supplementary Material 1 [file 12911_2026_3497_MOESM1_ESM.pdf]

Supplementary Table 1: Table comparing the performance of the model when different input image sizes are used during training on the Guangzhou dataset.

X-ray resolution	Accuracy	Accuracy_ std	Auc	Auc_std	Precision	Recall
256x256	48.0	0.17	92	0.02	94	37
600x600	84.0	0.08	99	0.01	83	99
768x768	58.0	0.18	95	0.01	89	58

Supplementary Table 2: Table comparing the performance of the model when different input patch sizes are used during training on the Guangzhou dataset.

Patch size	Accuracy
10x10	90.13
15x15	92.62
25x25	98.07
32x32	96.26
48x48	96.36

Supplementary Table 3: Table showing the performance of the model during the 5-fold cross validation during training on the Guangzhou dataset

Fold	Accuracy (%)	AUROC(%)	Precision(%)	Recall (%)
Fold 1	99.98	99.99	99.98	99.97
Fold 2	99.98	99.98	99.97	99.99
Fold 3	99.98	99.98	99.99	99.98
Fold 4	99.98	99.97	99.96	99.95
Fold 5	99.93	99.97	99.95	99.96
Standard Deviation	0.0224	8.367E-05	0.000158	0.000158
Standard Error	0.01	3.742E-05	7.1E-05	7.1E-05

Supplementary Table 4: Table comparing the performance of the model when different input image sizes are used during training on the Guangzhou dataset.

Fold	Validation Accuracy (%)	F1 Score	AUROC	Precision	Recall
1	99.99	0.9994	0.9993	0.9997	0.9991
2	99.98	0.9993	0.9992	0.9996	0.9990
3	99.98	0.9992	0.9991	0.9996	0.9989
4	99.98	0.9992	0.9991	0.9995	0.9988
5	99.93	0.9989	0.9988	0.9991	0.9987

Supplementary Table 5 — Table giving the confusion matrix

	**Pred NORMAL**	**Pred PNEUMONIA**	**Row Total**
**True NORMAL**	1493	32	1525
**True PNEUMONIA**	177	1348	1525
**Column Total**	1670	1380	3050

Supplementary Table 6: Performance of the model in the test set from the Guangzhou Women and Children's Medical Center chest X-ray dataset

**Performance metrics**	**Grid search(%)**	**5-fold cross validation(%)**	**5-fold SD**
Accuracy(ACC)	99	99	0.000224
Precision(P)	99	99	0.000158
Recall(SE)	99	99	0.000158
Specificity(SP)	99	99	N/A
F1 score	99	99	0.000141
AUROC score	99	99	N/A

Note: SD values are computed across five cross-validation folds. Specificity SD not shown (per-fold values unavailable). F1 SD computed from per-fold Precision and Recall as the harmonic mean.

Supplementary Table 7 - Table showing the architecture

**#**	**Layer**	**Type**	**Filters/Units**	**Kernel**	**Stride/Pool**	**Activation**
1	Input	Input	-	-	-	-
2	Conv1	Conv2D	32	3*3	1	ReLU
3	BN1	BatchNorm	-	-	-	-
4	Act1	Activation	-	-	-	ReLU
5	ResBlock1/ConvA	Conv2D	32	3*3	1	ReLU
6	ResBlock1/BN_A	BatchNorm	-	-	-	-
7	ResBlock1/ConvB	Conv2D	32	3*3	1	-
8	ResBlock1/BN_B	BatchNorm	-	-	-	-
9	ResBlock1/Add	Add (skip)	-	-	-	ReLU
10	MaxPool1	MaxPool2D	-	2*2	2	-
11	ResBlock2/Proj	Conv2D (1√ó1)	64	1*1	1	-
12	ResBlock2/Proj_BN	BatchNorm	-	-	-	-
13	ResBlock2/ConvA	Conv2D	64	3*3	1	ReLU
14	ResBlock2/BN_A	BatchNorm	-	-	-	-
15	ResBlock2/ConvB	Conv2D	64	3*3	1	-
16	ResBlock2/BN_B	BatchNorm	-	-	-	-
17	ResBlock2/Add	Add (skip)	-	-	-	ReLU
18	MaxPool2	MaxPool2D	-	2*2	2	-
19	ResBlock3/Proj	Conv2D (1√ó1)	128	1*1	1	-
20	ResBlock3/Proj_BN	BatchNorm	-	-	-	-
21	ResBlock3/ConvA	Conv2D	128	3*3	1	ReLU
22	ResBlock3/BN_A	BatchNorm	-	-	-	-
23	ResBlock3/ConvB	Conv2D	128	3*3	1	-

24	ResBlock3/BN_B	BatchNorm	-	-	-	-
25	ResBlock3/Add	Add (skip)	-	-	-	ReLU
26	GlobalAvgPool	GlobalAvgPool2D	-	-	-	-
27	Dense1	Dense	128	-	-	ReLU
28	Dropout	Dropout	-	-	-	-
29	Classifier	Dense	2	-	-	Softmax

Supplementary method

Architecture of the presented model:

Residual-network-based architecture: The image showcases a single convolutional neural network (CNN) architecture branch for simplicity, even though we have implemented two branches with the same components. The architecture has the following sections:

(1) **Initial Layers:** The initial processing begins with a convolutional layer named "initial layer consists of 1 SeparableConv2D layer, 1 BatchNormalization layer, and 1 RELU layer. The network begins with an **initial layer consisting of one SeparableConv2D layer, one BatchNormalization layer, and one ReLU activation layer**.

(2) **Residual layers:**

The architecture consists of an initial SeparableConv2D layer, batch normalization, and a ReLU activation. This is followed by five residual blocks, each incorporating SeparableConv2D layers and batch normalization. The final classification is performed using a concatenated softmax output layer.

The initial layer has been followed by 5 blocks of convolutional layer named conv1, conv2, conv3x, conv4, and conv5 with residual arrangement in each block (named as ResidualLayer 1-5. These blocks serve as the building blocks of the network, progressively learning intricate representations from the input data. Each block consists of 3 SeparableConv2D layers, 3 batch normalization layers, 2 ELU activation functions, 1 RELU activation function, 1 max pooling layer, and 1 additional layer for residual design. In the "conv5x" block the maximum pooling layer is replaced by a global pooling layer.

The convolution layers (conv1-5) utilize 64, 128, 256, 512, and 896 filters respectively, each with a kernel size of 7x7 pixels, and apply a stride of 2 during convolution. The resulting output

from the "convolution layer" has dimensions of 112x112 pixels but maintains a single-channel representation. The max-pooling layer reduces the spatial dimensions of the image to 56x56 pixels while preserving the single channel of data. As the network progresses through these blocks, the spatial dimensions further decrease based on the stride values. However, the single-channel representation is consistently maintained, ensuring a uniform feature representation throughout the network. This downsampling emphasizes the most critical features.

(3) **Final Layers**: The final layer consists of 1 additional SeparableConv2D and 1 ReLU layer.

The 2 branches together consist of 120 layers. The outputs from the two branches are concatenated using a concatenated layer. Next, a ReLU activation of the concatenated output followed by a softmax will provide the prediction output as healthy or pneumonia.
